# Mixed hexose and pentose sugars induce species-variable bacterial cellulose production by *Komagataeibacter* spp

**DOI:** 10.1007/s00449-025-03283-6

**Published:** 2026-01-28

**Authors:** Moyinoluwa O. Akintunde, Bukola C. Adebayo-Tayo, Obinna M. Ajunwa

**Affiliations:** 1 Institute for Sustainable Polymers and Composites, Aalen University, Aalen, Germany; 2 Department of Biological Sciences, KolaDaisi University, Ibadan, Nigeria; 3 Department of Microbiology, University of Ibadan, Ibadan, Nigeria; 4 Interdisciplinary Nanoscience Center (iNANO), Aarhus University, Aarhus, Denmark

**Keywords:** *Komagataeibacter*, Bacterial cellulose, Mixed carbon sources, Degree of crystallinity

## Abstract

Low-cost substrates and agricultural wastes for bacterial cellulose (BC) production have gained attention for their potential to increase yield and reduce costs. Diverse bacterial species exhibit heterogeneous metabolic profiles and substrate utilization patterns during BC biosynthesis on these substrates. This study aimed to determine the effects of hexose and pentose composition on BC yield by examining substrate utilization patterns of two *Komagataeibacter* species cultivated on mixed carbon sources. Cultivations were conducted over 16 days, with the sugar consumption pattern and BC yields determined. The produced BC was characterized using scanning electron microscopy (SEM), Fourier-transform infrared spectroscopy (FTIR), and X-ray diffraction (XRD). Both strains utilized mixed hexose and pentose sugars, but with distinct consumption patterns and yields. The highest BC yield (283%) was obtained by *Komagataeibacter* sp. CCUG73629 in glucose–cellobiose medium (M4), representing a 2.8-fold increase relative to the glucose-only medium (M6), while maximum substrate consumption (97.2%) was observed in glucose-only medium (M6) for *Komagataeibacter* sp. CCUG73630. FTIR showed characteristic cellulose peaks at 1163 and 1053 cm⁻¹, and SEM revealed densely interwoven fiber networks. XRD patterns displayed distinct peaks at 16.9° and 26.6°, with the highest crystallinity (67.5%) found in BC from *Komagataeibacter* sp. CCUG73630 grown in glucose–arabinose–xylose medium (M1). These findings indicate that each *Komagataeibacter* strain exhibits unique metabolic capacities and substrate utilization strategies. The study highlights the complexity and strain-specific nature of BC biosynthesis by each *Komagataeibacter* on mixed sugars and supports the development of efficient, economical methods for BC production for diverse industrial applications.

## Introduction

Bacterial cellulose (BC) is a polymeric material of microbial origin. It is a macromolecule with a wide range of applications [1]. With the rise in environmental challenges associated with the production and application of synthetic polymers, natural materials that can replace fossil-based polymers are constantly being developed. BC is an eco-friendly biomaterial that can replace fossil-based materials. BC although chemically identical to plant cellulose, composed of $\:\beta\:$-D-glucopyranose units linked by $\:\beta\:$-1,4 glycosidic bonds [2], is a purer form free of lignin and hemicellulose. It is characterized by a high degree of polymerization and crystallinity, and by a nanofibrillar network that provides a large surface-to-volume ratio [3]. These properties give BC higher strength, water-holding capacity and biocompatibility compared to plant cellulose, favouring its application in biomedical devices, tissue engineering, cosmetics and sensors, while plant cellulose remains dominant in large scale, low-cost applications such as paper, textile and construction. BC is produced as an extracellular polymer by diverse groups of microorganisms, with acetic acid bacteria being the most promising. Bacteria of the genera *Acetobacter*,* Komagataeibacter*,* Rhizobium*,* Pseudomonas*,* Agrobacterium*,* Sarcina* and others have the ability to produce BC [4]. *Komagataeibacter* species such as *K. europaeus*,* K. hansenii*,* K. rhaeticus*,* and K. xylinus* are among the best producers of BC and can utilize different carbon sources [5, 6].

Several factors contribute to the efficient production of BC, including the carbon source and pH of the cultivation media. The carbon source is one of the most extensively studied components of the growth medium, as it significantly affects the BC yield [1]. However, carbon sources contribute to the high cost of producing BC, which has led to research on alternative low-cost substrates. Several works have focused on the use of low-cost, available wastes as substrates for BC production. Low-cost carbon sources such as juices, peels and pomaces of orange, pineapple, and pawpaw have been previously studied for BC production [6–10]. Agricultural residues such as sugarcane bagasse, corn cobs, corn stalks, rice straw, and durian shells have also been used for BC production [9, 11–13]. These agricultural residues are mostly lignocellulosic materials, which, when pretreated, contain significant quantities of both hexose sugars and pentose sugars, such as xylose, arabinose, and glucose, which are major carbon sources. BC-producing bacteria consume these sugars at different rates and utilize them for BC production, resulting in different BC yields, with glucose consistently reported as the most efficient substrate, whereas pentoses such as xylose and arabinose often result in reduced yields [7]. In *Komagataeibacter* spp., BC biosynthesis depends on metabolic processes that govern precursor availability, including glucokinase activity, hexose and pentose transport systems, and the routing of intermediates into UDP-glucose, the precursor for cellulose synthase [5]. During BC synthesis with glucose as the sole carbon source, glucose directly enters glycolysis and feeds precursor (UDP-glucose), however, other hexoses (e.g., fructose, mannose) and disaccharides (e.g., sucrose, cellobiose) often require additional enzymatic steps, which can reduce carbon flux towards BC or lead to by‑products that inhibit synthesis [5]. Pentoses such as xylose and arabinose, abundant in lignocellulosic hydrolysates [13], are utilized less efficiently because their assimilation relies on specific isomerases and kinases and proceeds via the pentose phosphate pathway, which is a more energy-consuming process. These metabolic limitations help explain the substrate‑specific differences in BC yield reported in earlier studies. The objective of this study was to mimic the hexose and pentose sugar composition of low-cost substrates, assuming that the substrate is made up of at least one hexose sugar and one pentose sugar for BC production, which will provide better insight into the pattern of sugar consumption by strains during BC production. In this study, some hexose and pentose sugars were combined at different ratios as carbon sources for BC production by *Komagataeibacter* spp., and the rates of individual sugar consumption and the effects of mixed carbon sources on BC yield and crystallinity were studied.

## Methods

### Bacterial cellulose (BC) production

Two BC-producing *Komagataeibacter* strains previously isolated by Akintunde et al. [13], identified as *Komagataeibacter* sp. CCUG73629 and *Komagataeibacter* sp. CCUG73630, were sub-cultured on Hestrin-Schramm (HS) media g/L (composed of yeast extract-5; glucose-20; peptone-5, citric acid-1.15, disodium hydrogen phosphate-2.7, and agar‒agar-15) and incubated at 30 °C for 3–5 days [14]. One loopful of colonies from the agar plates were transferred into HS broth and incubated for 3 days at 30 °C to develop the seed inoculum for BC production.

### Effect of a mixed carbon source on BC production

To study the effects of mixed carbon sources on BC production, the two *Komagataeibacter* strains were cultivated in HS media supplemented with combination of hexose and pentose sugars as carbon source (Table 1). The total concentration of hexose and pentose sugars in each media was maintained at 28 g/L, while the proportion of each sugar varied among the media formulations. The effects of the media composition on the BC yield, final pH of the fermentation media and substrate consumption by the isolates were determined. The pH of the media was adjusted to 6.0. After sterilization, 5 mL of the seed inoculum was inoculated into 100 mL of the fermentation media and incubated statically for 16 days at 30 °C. After 16 days of fermentation, the quantity of BC produced, the amount of substrate consumed and the final pH of the media were determined.

**Table 1:** Media composition with different sugar combinations used as carbon sources for BC production

Sugars	M1 + HS components	M2 + HS components	M3 + HS components	M4 + HS components	M5 + HS components	M6 + HS components
Glucose	+	+	+	+	+	+
Arabinose	+	+	+			
Xylose	+	+	+		+	
Cellobiose		+		+		
Mannose			+			
Galactose			+			

Key: M1: Medium 1, M2: Medium 2, M3: Medium 3, M4: Medium 4, M5: Medium 5, M6: Medium 6

### BC purification, pH and substrate consumption determination

The BC produced after fermentation was harvested by picking with tweezers. BC purification was performed by boiling for 1 h at 80 °C in 1 M NaOH to remove cells and medium residue [13]. Thereafter, the BC was washed with distilled water until it reached pH 7 and allowed to air dry under ambient conditions until a constant weight was achieved. The dry weight of BC (grams per liter of fermentation media (g/L)) was measured via an analytical weighing balance (Kern PFB precision balance, Sigma Aldrich).

To facilitate comparison across different media compositions, the BC yields in this study were expressed relative to the dry weight of BC obtained in the glucose-only (M6) medium, which served as the reference condition. The relative yield (%) was calculated as follow in Eq. [1]:

1 $$\:\mathrm{B}\mathrm{C}\:\mathrm{y}\mathrm{i}\mathrm{e}\mathrm{l}\mathrm{d}\:\left(\mathrm{\%}\right)\:=\:\frac{BC\:dry\:weight\:in\:mixed\:carbon\:source\:(g/L)}{BC\:produced\:in\:glucose\:only\:medium\:(g/L)\:}\times\:100$$

The final pH of the fermentation media after BC production was measured with a pH meter. The rate of substrate consumption was determined via high-performance liquid chromatography (HPLC) (Walters Corporation, Milford, USA) by measuring the concentrations of various sugar components in the fermentation media. One milliliter of the fermentation media was pipetted into Eppendorf tubes and centrifuged for 10 min at 7000 rpm. A 0.2 μm filter was used for filtration of the supernatant into HPLC vials. A hydrogen base ion-exchange column (Aminex HPX-87 H, Bio-Rad, Hercules, USA) that works at 60 °C, with 5 mM H_2_SO_4_ solution as the eluent, flowing at 0.6 mL/min, was used for the detection and quantification of sugars. The substrate consumption according to [13] was calculated as follows in Eq. [2]:

2 $$\begin{aligned}&\mathrm{S}\mathrm{u}\mathrm{b}\mathrm{s}\mathrm{t}\mathrm{r}\mathrm{a}\mathrm{t}\mathrm{e}\:\mathrm{c}\mathrm{o}\mathrm{n}\mathrm{s}\mathrm{u}\mathrm{m}\mathrm{p}\mathrm{t}\mathrm{i}\mathrm{o}\mathrm{n}\:\left(\mathrm{\%}\right)\\ &\quad= \frac{Initial\:total\:sugar\:-Final\:total\:sugar}{Final\:Total\:sugar}\:\times\:100\:\end{aligned}$$

### Scanning electron microscopy (SEM)

The morphology of the BC produced by the bacterial strains was determined using SEM. Dried BC films were placed on a carbon tape, thereafter coated with gold using LICA EM ACE 600 (Leica, Germany). The gold coated samples were observed with a scanning electron microscope SEM LEO Gemini 1525 (Carl Zeiss, Germany), operated at an accelerating voltage of 5.00 kV, a working distance (WD) of 8.6 mm and a magnification of 15,000x.

### Fourier transform infrared (FTIR) spectroscopy

FTIR was performed via an FTIR spectrometer (Shimadzu model, Germany). The BC samples were analysed by placing the dried film on a diamond accessory. With an accumulation of 128 scans, the absorbance was measured in the wavenumber range of 4000 –500 cm^− 1^.

### X-ray diffraction (XRD)

XRD was performed to determine the degree of crystallinity of the BC. X-ray diffraction analysis was performed by measuring the diffraction pattern of the BC sheet via a diffractometer (Siefert Sun XRD 3003) operating at 40 kV and 40 mA. The diffractograms were taken from 0 to 60° on a 2θ scale with a step size of 0.02°. The determination of the crystallinity index was based on the method described by Segal et al. [15]. The determination of the crystalline and amorphous content of BC described below, where $\:{I}_{200}$ is the maximum intensity of the (200 diffraction peak and $\:{I}_{am}$ is the intensity at the amorphous minimum is described in Eq. [3].

3 $$\begin{gathered} {\mathrm{Crystallinity~Index}}~\left( {CI} \right)=~\frac{{{I_{200}} - {I_{am}}}}{{{I_{200}}}}~ \times 100\% \hfill \\ {\mathrm{Amorphous~content~}}=100\% ~ - CI \hfill \\ \end{gathered}$$

### Statistical analysis

All the experiments were carried out in duplicate, and the values are presented as the means ± SDs. All the data were statistically analysed via MINITAB 17. The data were evaluated via One-Way Analysis of Variance (ANOVA) and Tukey test was used to determine the significant difference between groups.

## Results

### Effect of the media composition on the BC yield, final pH and total substrate consumption

The effects of the media composition on the BC yield, final pH and substrate consumption of the fermentation media by *Komagataeibacter* sp. CCUG73629 and *Komagataeibacter* sp. CCUG73630 are shown in Tables 2 and 3. The BC yield and final pH of the media enriched with *Komagataeibacter* sp. CCUG73629 ranged from 100 to 283.3% and 3.7–4.2, respectively. The highest yield was recorded in M4, whereas the lowest BC yield was observed in M6. The highest pH was observed in M2, and the lowest was observed in M6. For *Komagataeibacter* sp. CCUG73630, the highest BC yield and final pH of the media ranged from 43.5 to 100.0% and 3.5–4.0, respectively. The highest yield was recorded in M6, whereas the lowest yield was recorded in M3. The highest pH was observed in M2, and the lowest was observed in M5. During BC production, the highest total substrate consumption of 81.5% was observed in M3 (glucose, mannose, xylose, galactose and arabinose) by *Komagataeibacter* sp. CCUG73629, whereas in *Komagataeibacter* sp. CCUG73630, a total substrate consumption of 97.2% was observed in M6 (glucose). However, in M5 (glucose and xylose), 82.6% of the substrate was consumed by *Komagataeibacter* sp. CCUG73630.

**Table 2:** Effects of media composition on BC yield, substrate consumption and pH by *Komagataeibacter* sp. CCUG73629

Mixed carbon Media	BC dry weight (g/L)	BC yield (%)	Total substrate consumption (%)	pH
M1	0.84 ± 0.05^bc^	211.0 ± 1.41^c^	75.2 ± 1.10^b^	3.8 ± 0.01^c^
M2	0.91 ± 0.13^ab^	228.3 ± 1.06^b^	55.4 ± 1.08^d^	4.2 ± 0.01^a^
M3	0.48 ± 0.01^cd^	119.5 ± 0.71^e^	81.5 ± 1.59^a^	4.0 ± 0.28^b^
M4	1.13 ± 0.31^a^	283.3 ± 1.06^a^	62.6 ± 1.27^c^	3.8 ± 0.01^c^
M5	0.7 ± 0.13^bc^	176.5 ± 2.12^d^	77.1 ± 1.28^ab^	3.8 ± 0.01^c^
M6	0.4 ± 0.01^d^	100.0 ± 0.00^f^	56.4 ± 1.12^d^	3.7 ± 0.01^d^

Keywords: M1 Glucose + Arabinose + Xylose, M2 Glucose + Cellobiose + Xylose + Arabinose, M3: Glucose + Mannose + Xylose + Galactose + Arabinose, M4 Glucose + Cellobiose, M5 Glucose + Xylose, M6 Glucose

The letters indicate which groups are significantlly different from one another. This is part of the statistical analysis

**Table 3:** Effects of media composition on BC yield, substrate consumption and pH by *Komagataeibacter* sp. CCUG73630

Mixed carbon Media	BC Dry weight (g/L)	BC yield (%)	Total substrate consumption (%)	pH
M1	0.46 ± 0.08^ab^	68.8 ± 0.17^e^	76.9 ± 0.01^c^	3.7 ± 0.03^c^
M2	0.52 ± 0.14^ab^	77.8 ± 0.28^d^	64.9 ± 0.16^d^	4.0 ± 0.03^a^
M3	0.29 ± 0.05^b^	43.5 ± 0.27^f^	60.0 ± 0.27^e^	3.8 ± 0.01^b^
M4	0.61 ± 0.16^a^	91.0 ± 0.03^b^	76.3 ± 0.22^c^	3.6 ± 0.03^c^
M5	0.57 ± 0.04^a^	85.1 ± 0.02^c^	82.6 ± 0.40^b^	3.5 ± 0.01^d^
M6	0.67 ± 0.03^a^	100.0 ± 0.00^a^	97.2 ± 0.12^a^	3.7 ± 0.01^c^

Keywords: M1 Glucose + Arabinose + Xylose, M2 Glucose + Cellobiose + Xylose + Arabinose, M3 Glucose + Mannose + Xylose + Galactose + Arabinose, M4 Glucose + Cellobiose, M5 Glucose + Xylose, M6 Glucose

The letters indicate which groups are significantlly different from one another. This is part of the statistical analysis

### Substrate consumption pattern of individual hexose and Pentose sugars by *Komagataeibacter* sp. CCUG73629 and *Komagataeibacter* sp. CCUG73630 during BC production in mixed carbon sources

**Fig. 1:**
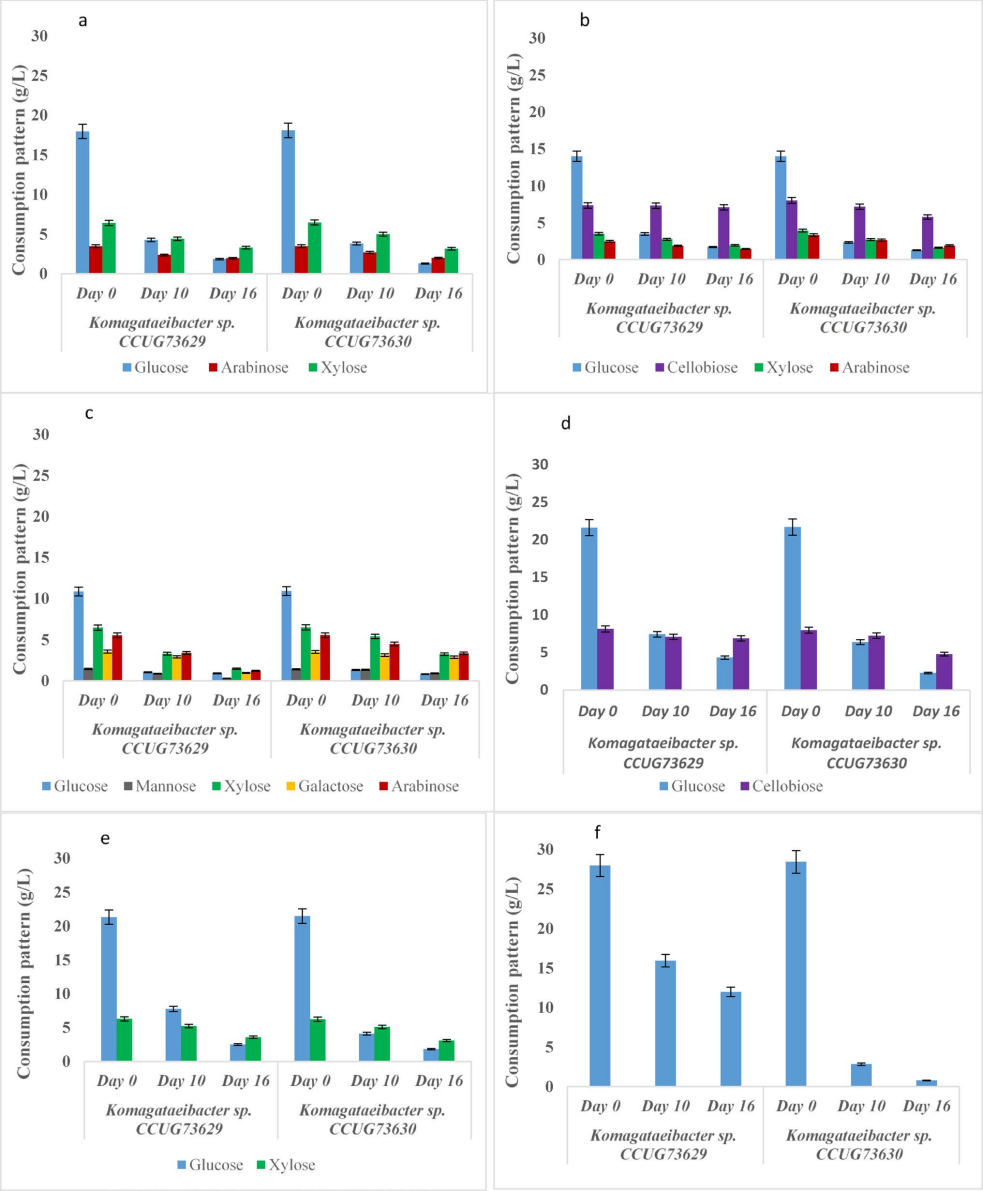
**a**–**f** Consumption patterns of *Komagataeibacter* sp. CCUG73629 and *Komagataeibacter* sp. CCUG73630 in mixed carbon media: **a** M1, **b** M2, **c** M3, **d** M4, **e** M5, and **f** M6 during BC production

The pattern of substrate consumption by *Komagataeibacter* sp. CCUG73629 and *Komagataeibacter* sp. CCUG73630 in the mixed carbon source medium during BC production is shown in Fig. 1a–f. During BC production by *Komagataeibacter* sp. CCUG73629 in M1, the consumption of glucose, arabinose and xylose ranged from 18.0 to 1.9 g/L, 3.5–1.9 g/L and 6.4–3.3, respectively. By day 16, 89% of the glucose had been consumed. During BC production by *Komagataeibacter* sp. CCUG73630 in M1, the consumption of glucose, arabinose and xylose ranged from 18 –1.3 g/L, 3.5–1.9 g/L and 6.5–3.2 g/L, respectively. By day 16, 92% of the glucose and 50% of the xylose were consumed.

In M2, during BC production by *Komagataeibacter* sp. CCUG73629, the consumption of glucose, cellobiose, xylose and arabinose ranged from 14 –1.7 g/L, 7.4–7.1 g/L, 4–1.9 g/L and 2.5–1.5 g/L, respectively. After 16 days, 87% of the glucose and 3% of the cellobiose were consumed. During BC production by *Komagataeibacter* sp. CCUG73630 in M2, the consumption of glucose, cellobiose, xylose and arabinose ranged from 14 –1.3 g/L, 8–5.8 g/L, 3.9–1.6 g/L and 3–1.9 g/L, respectively. By the 16th day, 91% of the glucose, 27% of the cellobiose and 58% of the xylose were consumed.

During BC production via M3 and *Komagataeibacter* sp. CCUG73629, the consumption of glucose, mannose, xylose, galactose and arabinose ranged from 10.8 to 0.9 g/L, 1.4 –0.3 g/L, 6.4–1.4 g/L, 3.5–0.9 g/L and 5.5–5.5 g/L, respectively. After 16 days, 91% of the glucose and over 70% of the other sugars were consumed. During BC production by *Komagataeibacter* sp. CCUG73630, glucose, mannose, xylose, galactose and arabinose consumption ranged from 10.8 to 0.8 g/L, 1.4–0.9 g/L, 6.5–3.2 g/L, 3.5–2.9 g/L and 5.5–3.3 g/L, respectively. After 16 days, 92% of the glucose, 50% of the xylose and 18% of the galactose were consumed.

With the use of M4 during BC production by *Komagataeibacter* sp. CCUG73629, the consumption of glucose and cellobiose ranged from 21.6 to 4.3 g/L and 8.1–6.9 g/L, respectively. During production by *Komagataeibacter* sp. CCUG73630, the consumption of glucose and cellobiose ranged from 21.7 to 2.3 g/L and 8.0–2.8 g/L, respectively. After 16 days, *Komagataeibacter* sp. CCUG73629 consumed 79% of the glucose and 15% of the cellobiose, whereas *Komagataeibacter* sp. CCUG73630 consumed 89% of the glucose and 39% of the cellobiose.

During BC production with M5, *Komagataeibacter* sp. CCUG73629, the consumption of glucose and xylose ranged from 21.3 to 2.4 g/L and 6.2–3.5 g/L, respectively, whereas the consumption of glucose and xylose by *Komagataeibacter* sp. CCUG73630 ranged from 21.4 to 1.8 g/L and 6.2–3.0 g/L, respectively. After 16 days of BC production, 88% of the glucose and 43% of the xylose were consumed by *Komagataeibacter* sp. CCUG73629, whereas 91% of the glucose and 50% of the xylose were consumed by *Komagataeibacter* sp. CCUG73630.

Glucose consumption in M6 ranged from 28 –12 g/L and 28.4–0.7 g/L by *Komagataeibacter* sp. CCUG73629 and *Komagataeibacter* sp. CCUG73630 during BC production. By the 16th day, *Komagataeibacter* sp. CCUG73629 consumed 57%, while *Komagataeibacter* sp. CCUG73630 consumed 97% of glucose.

### Characterization of BC produced with mixed carbon sources

**Fig. 2:**
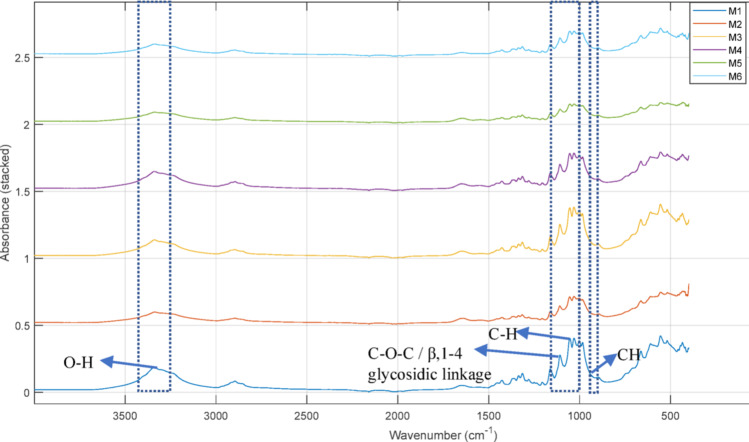
FTIR spectra of BC produced by *Komagataeibacter* sp. CCUG73629 in mixed carbon media

**Fig. 3:**
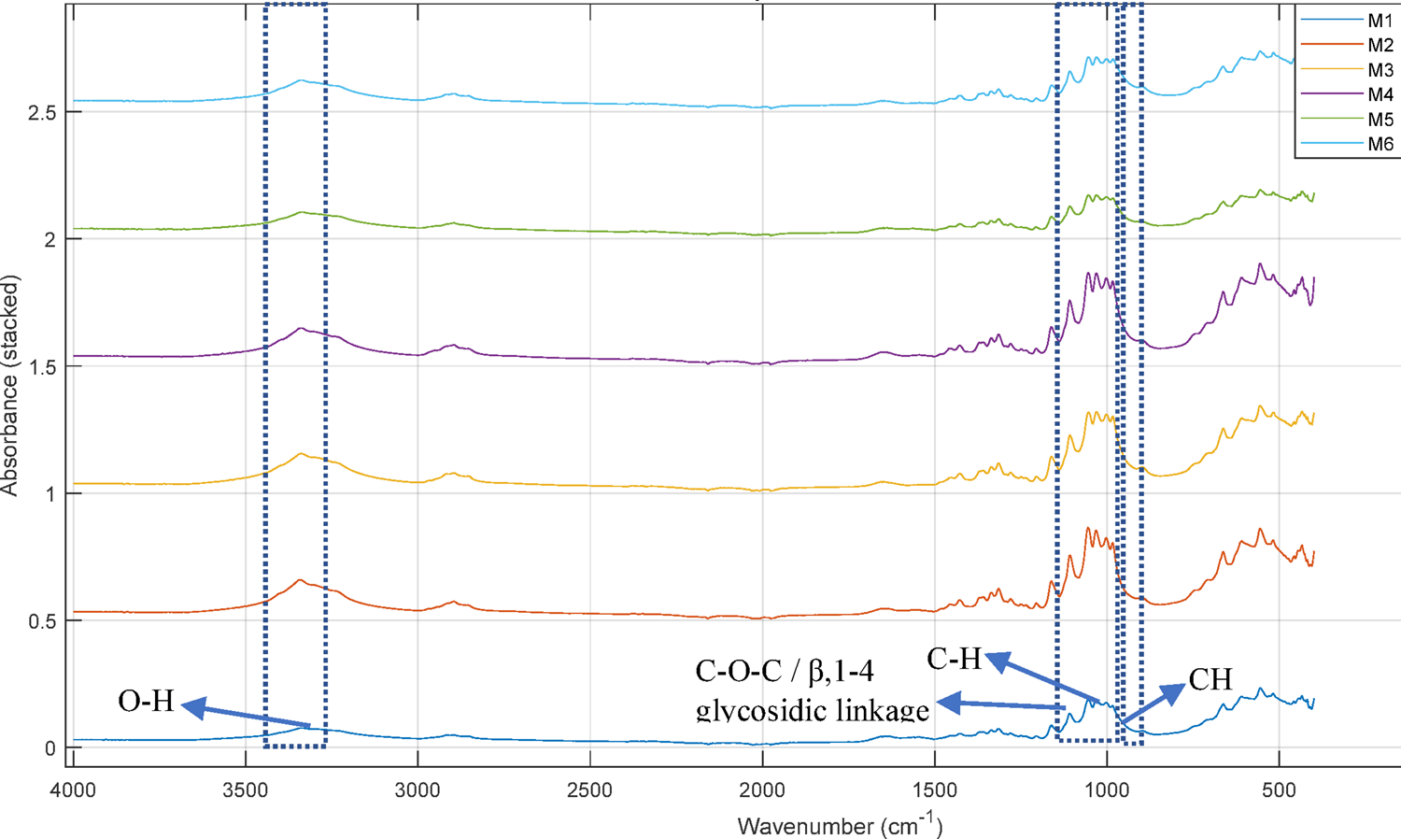
FTIR spectra of BC produced by *Komagataeibacter* sp. CCUG73630 in mixed carbon media

The chemical structures of the BCs produced from *Komagataeibacter* sp. CCUG73629 and *Komagataeibacter* sp. CCUG73630 in mixed carbon media are shown in the FTIR spectra in Figs. 2 and 3. The BCs produced by both strains were similar, with slight differences in peak positions and absorbances. The broad peaks at approximately 3338 and 3340 cm^− 1^ at both strains correspond to O-H stretching vibrations. The peaks at 2891 and 2895 cm^− 1^ indicate C-H group stretching. The peaks at approximately 1641 and 1651 cm^− 1^ correspond to O-H bending of the absorbed water. The peaks at 1427 and 1429 cm^− 1^ are associated with CH_2_ bending. The peaks at 1163 and 1161 cm^− 1^ indicate asymmetrical C-O-C stretching. The peaks at approximately 1053 and 1055 cm^− 1^ indicate vibrations of C-C, C-OH and C-H rings and side groups. The peak at approximately 896 cm^− 1^ at both strains corresponds to CH vibrations.

**Fig. 4:**
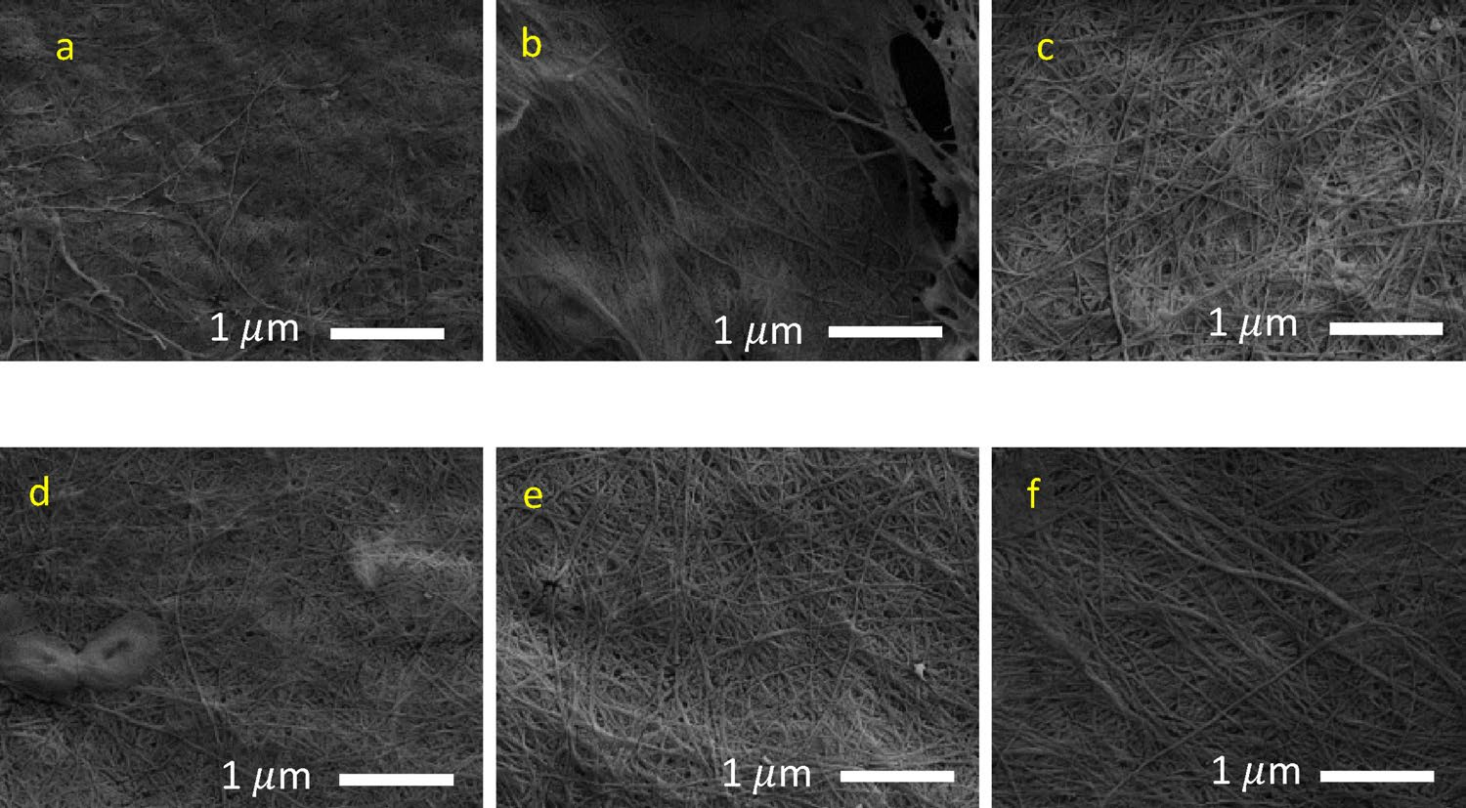
Micrograph of BC produced by *Komagataeibacter* sp. CCUG73629 (**a–c**) and *Komagataeibacter* sp. CCUG73630 (**d–f**) in M1, M4 and M6 at 15,000x magnification

The morphology of the BC produced by *Komagataeibacter* sp. CCUG73629 and *Komagataeibacter* sp. CCUG73630 in M1, M4 and M6 is shown in Fig. 4a–f. The BC micrograph showed a dense network of oriented fibers produced by both strains. The BC produced by *Komagataeibacter* sp. CCUG73629 in M1 and M4 was more compact and denser, with the fibres tightly aggregated, while the BC fibers produced by *Komagataeibacter* sp. CCUG73630 in M1 and M4 densely interwoven. In M6, both strains-produced similar BC fibers that were densely interwoven.

**Fig. 5:**
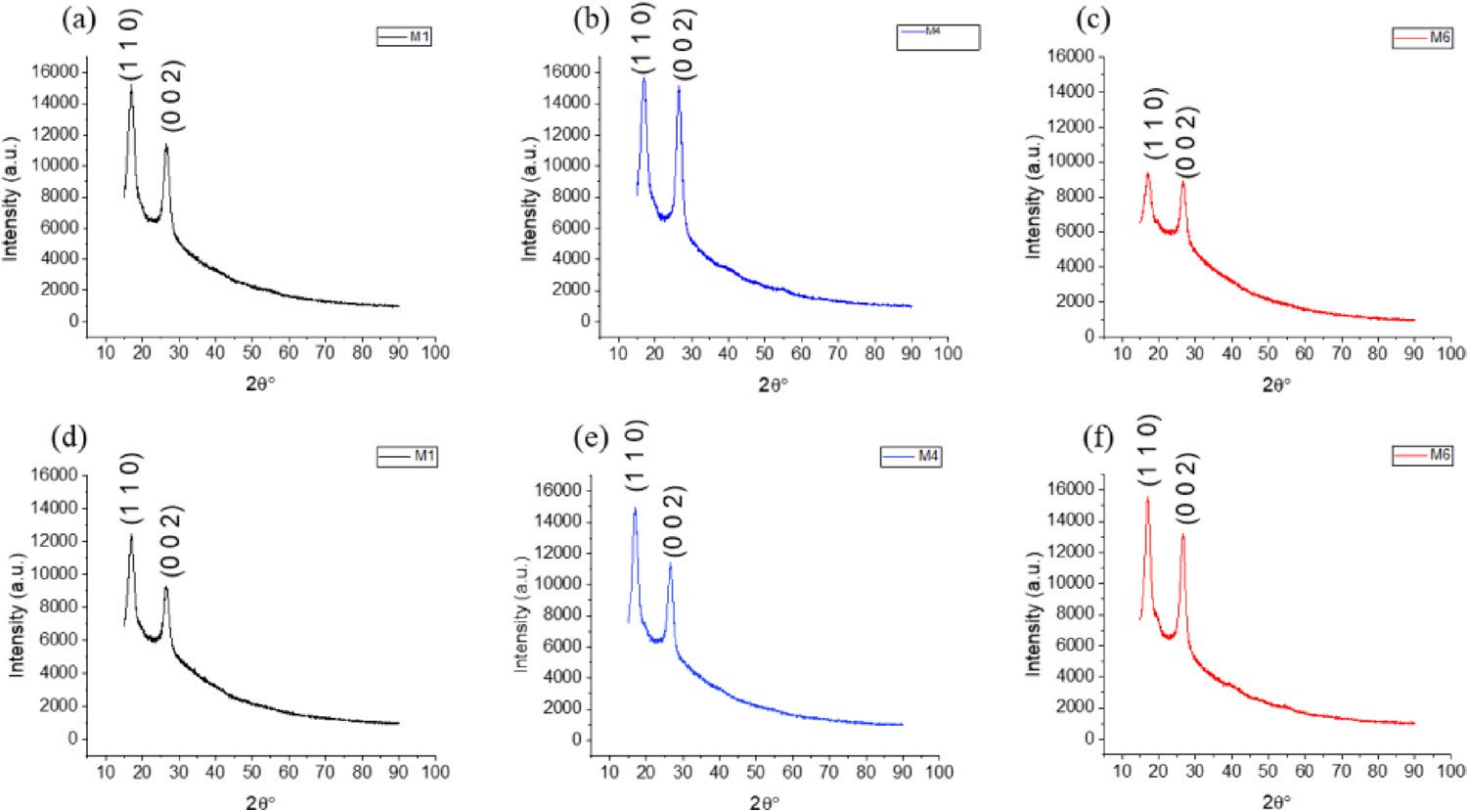
X-ray diffractogram of BC produced by *Komagataeibacter* sp. CCUG73629 (**a**–**c**) and *Komagataeibacter* sp. CCUG73630 (**d**–**f**) in M1, M4 and M6

The X-ray diffractograms of the BCs produced in M1, M4 and M6 presented two distinct characteristic peaks around 16.9$\:^\circ\:$ and 26.6$\:^\circ\:$ (Fig. 5). The degree of crystallinity and amorphous content of the BC varied with different carbon sources (Table 4). In M1, BC produced by *Komagataeibacter* sp. CCUG73630 had a greater degree of crystallinity (67.5%) than BC produced by *Komagataeibacter* sp. CCUG73629 (58.6%). However, the M6 BC produced by *Komagataeibacter* sp. CCUG73629 had a relatively high degree of crystallinity (66.7%).

**Table 4:** Degree of crystallinity and amorphous content of BC produced in mixed carbon media

	Komagataeibacter sp. CCUG73629		Komagataeibacter sp. CCUG73630	
BC produced in Mixed carbon media	Degree of crystallinity (%)	Amorphous content (wt%)	Degree of crystallinity (%)	Amorphous content (wt%)
BC_M1	58.6	41.4	67.5	32.5
BC_M4	60.2	39.8	61.2	38.8
BC_M6	66.7	33.2	58.1	41.9

Keywords: M1 − Glucose + Arabinose + Xylose; M4 − Glucose + Cellobiose; M6 – Glucose

## Discussion

**Fig. 6:**
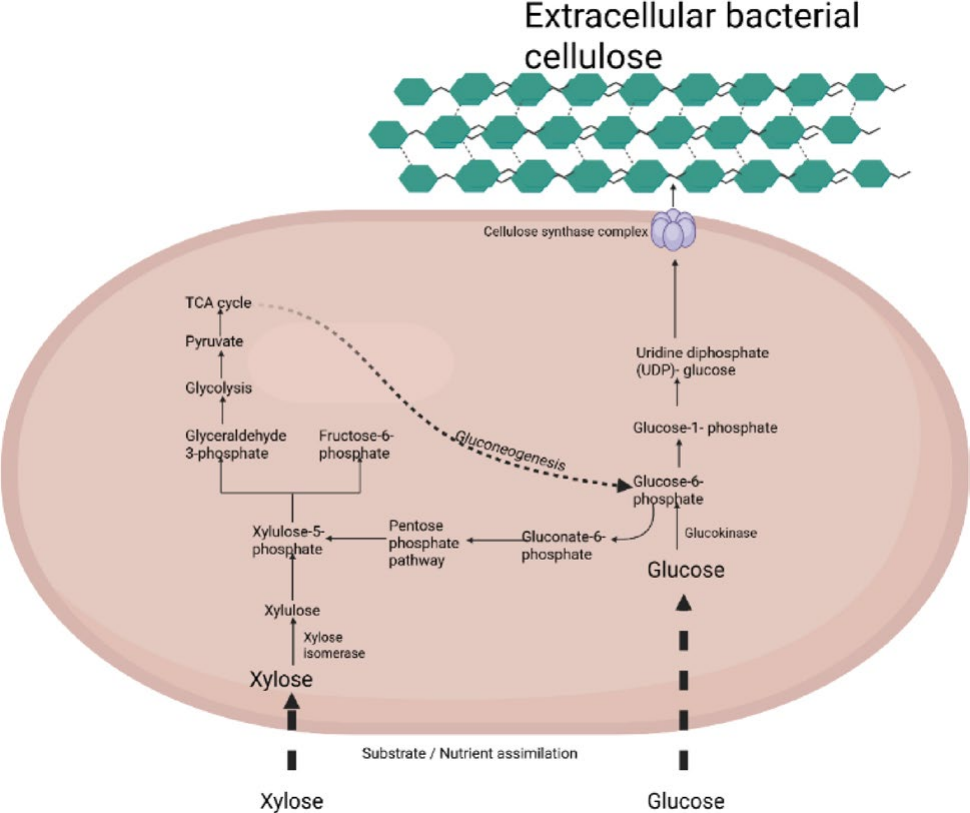
Generalised schematic representation of hexose and pentose metabolism for BC production. Created in BioRender. Ajunwa, O. (2025) https://BioRender.com/ejgy96y

The effect of media composition on BC production provides insight into how bacteria can utilize mixed hexose and pentose as a carbon source for production. Growth media containing different sources of nutrients are important for determining the yield and characteristic properties of BC produced by different bacterial species. The carbon source is vital for BC production, as yield depends on the availability and quality of the carbon source [16]. During BC synthesis (Fig. 6), hexose sugars are directly metabolized via glycolysis to produce glucose-6-phosphate, a precursor for UDP-glucose, for BC synthesis, which is energy efficient, enabling a relatively high BC yield. However, pentose sugars enter the pentose phosphate pathway, requiring gluconeogenesis to generate BC precursors and consuming more ATP, which may reduce BC production efficiency [17]. *Komagataeibacter* species among the acetic acid bacteria are widely studied for BC production because they achieve considerable BC yields and metabolize different carbon sources [18, 5]. The total substrate consumption did not significantly affect the BC yield. Increased substrate consumption may not always correspond to increased BC yield, because, some of the carbon may be directed towards cell survival or physiological adjustment rather than BC synthesis. During BC production by *Komagataeibacter* sp. CCUG73629, the medium that contained glucose as the sole source of carbon least supported BC production, which could indicate that the bacterial cells thrived better in media containing more than one carbon source. This may be a result of catabolite repression in single sugar media due to the relatively high concentration of a single sugar (glucose), which can inhibit enzymatic reactions involved in sensing endogenous levels of sugars and can be avoided when mixed sugars are utilized [19, 20]. Zhong et al. [21] suggested that the production of BC may be efficient by adding a combination of several carbon sources, as they reported increased BC production in media containing glucose, glycerol and fructose as mixed carbon sources. When *Komagataeibacter* sp. CCUG73630 was used for BC production, the glucose-only medium supported the highest BC yield, which means that a single carbon source was more efficient for BC production than a mixed carbon source was. *Komagataeibacter* sp. CCUG73630 also consumed more glucose than did *Komagataeibacter* sp. CCUG73629, indicating that *Komagataeibacter* sp. CCUG73630 preferred glucose as the sole carbon source for BC production. With glucose as the carbon source, both strains presented similar yields of BC, although glucose consumption was lower in *Komagataeibacter* sp. CCUG73629. During BC production, glucose can be used as a precursor for the assembly of glucose units into cellulose [22]. The medium containing glucose and cellobiose as the carbon source supported the highest BC production by *Komagataeibacter* sp. CCUG73629, with only 15% of cellobiose consumed, whereas M4 had the second-best BC yield by *Komagataeibacter* sp. CCUG73630, 39% of the cellobiose was consumed. These findings emphasize the metabolic versatility of the individual strains in the medium, indicating that cellobiose may have the ability to activate BC production even though consumption was low, making cellobiose an inducer of BC production. Qi et al. [23] reported that after the 2nd day of fermentation, when little cellobiose was consumed, *Gluconacetobacter xylinus* could not further consume cellobiose during BC production.

The medium with the most diverse sugar mixture containing glucose, mannose, xylose, galactose and arabinose resulted in the lowest BC production for both *Komagataeibacter* sp. CCUG73629 and *Komagataeibacter* sp. CCUG73630, with both having similar total substrate consumption. However, when Dahman et al. [20] used *G. xylinus* ATCC700178, greater production was recorded for glucose, mannose, xylose, galactose and arabinose mixtures. The unique enzymatic sets and metabolic pathways of the individual strains can be a factor for the varied abilities to metabolize hexoses and pentoses. As explained in Fig. 6. Some strains will metabolize glucose directly and efficiently, via glycolysis, because glucose enters the central metabolism with minimal enzyme modification, which is not the same for pentose metabolism, which may require additional enzymatic steps or metabolic shifts.

The carbon sources used for BC production affect its properties, such as the crystallinity index [22]. The mixed carbon sources influenced the degree of crystallinity and amorphous content of the BC produced by both strains. The X-ray diffractograms of the BCs produced by both strains in the mixed carbon media were similar, with slight differences in intensity. The characteristic peaks were assigned to the (110) and (002) crystallographic planes, which corresponds to the cellulose I structure [24, 25]. The crystallinity of BC varied with the strain producing it and the medium. However, the crystallinity of BC produced by both strains in M4 was not significantly different. Chen et al. [26] reported that the crystallinity of BC produced in single sugar media (galactose, xylose and mannose) was greater than that of BC produced in mixed sugar media; however, the yield of BC was lower in single sugar media. The morphology of the BC produced by both strains showed only slight differences. The compact, densely interwoven structure of the BC fibres was similar to those in the study of Arooj et al. [27] who reported similar effect for BC produced by *Komagataeibacter* sp. LMG 18,909 and FXV3 in glucose and glycerol with 3.0% ethanol. There was no significant difference in the chemical structure of BC. The functional groups present were similar with those reported by Akintunde et al. [13] during BC production in agricultural residue. The FTIR spectra of both strains in mixed carbon media indicated that all the peaks were consistent with previous characterization of bacterial cellulose [28, 29]. The BC produced from the hexose-rich media exhibited higher crystallinity and dense interwoven fiber structures, making it desirable for biomedical application like in wound healing and drug delivery, with crystallinity been a critical property for applications requiring strength and durability.

## Conclusion

This study demonstrated the complexity and strain-specific nature of *Komagateibacter* sp. CCUG73629 and *Komagataeibacter* sp. CCUG73630 during BC production in mixed carbon sources. These distinctions stem from differences in their metabolic pathways, energy efficiency and regulatory mechanisms. The strains utilized the carbon sources in the media simultaneously at varying rates, with glucose being prioritized for initial consumption at the highest rate of up to 90% after 10 days followed by slower consumption (less than 50%) of other sugars in the medium. Efforts to enhance BC production using alternative carbon sources, including agricultural wastes and lignocellulosic feedstocks such as sugarcane bagasse, corncob, wheat straw, and rice straw, offer a sustainable approach. Hydrolysates of these materials typically contain mixed sugars; glucose, arabinose, xylose, galactose, mannose, and cellobiose. This study’s combination of sugars reflects these compositions, revealing distinct consumption patterns and their impact on BC yield and selected physicochemical properties. Such insights are essential for optimizing BC production for diverse future applications. Further research should focus on refining BC yields through genetic engineering of promising *Komagataeibacter* strains, investigating in greater depth the specific role of cellobiose in BC biosynthesis, and elucidating the physiological and metabolic mechanisms underlying sugar utilization in individual strains.

## Data Availability

The authors declare that the data supporting the findings of this study are available within the paper. Should any raw data files be needed in another format they are available from the corresponding author upon reasonable request.
